# Inpatient Virtual Vision Clinic Improves Access to Vision Rehabilitation Before and During the COVID-19 Pandemic

**DOI:** 10.1016/j.arrct.2020.100100

**Published:** 2020-12-19

**Authors:** Matthew Keilty, Kevin E. Houston, Caroline Collins, Ritika Trehan, Ya-Ting Chen, Lotfi Merabet, Amy Watts, Shrinivas Pundlik, Gang Luo

**Affiliations:** a Spaulding Rehabilitation Hospital Cape Cod, East Sandwich, Massachusetts; b Spaulding Rehabilitation Hospital, Cambridge, Massachusetts; c Department of Physical Medicine and Rehabilitation, Spaulding Rehabilitation Hospital, Harvard Medical School, Boston, Massachusetts; d Optometry and Vision Rehabilitation Service, Massachusetts Eye and Ear, Boston, Massachusetts; e Department of Ophthalmology, Harvard Medical School, Boston, Massachusetts; f Schepens Eye Research Institute, Boston, Massachusetts

**Keywords:** Pandemics, Rehabilitation, Telemedicine, Vision, ocular, app, application, AR, acute rehabilitation, COVID-19, coronavirus disease 2019, EOM, extraocular movement, IRF, inpatient rehabilitation facility, IQR, interquartile range, LTAC, long-term acute care, OD, Doctor of Optometry, OT, occupational therapist, SARS-CoV-2, severe acute respiratory syndrome coronavirus 2

## Abstract

**Objective:**

To describe and evaluate a secure video call system combined with a suite of iPad vision testing apps to improve access to vision rehabilitation assessment for inpatients.

**Design:**

Retrospective.

**Setting:**

Two acute care inpatient rehabilitation hospitals and 1 long-term acute care (LTAC) hospital.

**Participants:**

Records of inpatients seen by the vision service.

**Interventions:**

Records from a 1-year telemedicine pilot performed at acute rehabilitation (AR) hospital 1 and then expanded to AR hospital 2 and LTAC hospital during coronavirus disease 2019 (COVID-19) were reviewed. In the virtual visits, an occupational therapist measured the patients’ vision with the iPad applications and forwarded results to the off-site Doctor of Optometry (OD) for review prior to a video visit. The OD provided diagnosis and education, press-on prism application supervision, strategies and modifications, and follow-up recommendations. Providers completed the telehealth usability questionnaire (10-point scale).

**Main Outcome Measures:**

Vision examinations per month at AR hospital 1 before and with telemedicine.

**Results:**

With telemedicine at AR hospital 1, mean visits per month significantly increased from 10.7±5 to 14.9±5 (*P*=.002). Prism was trialed in 40% of cases of which 83% were successful, similar to previously reported in-person success rates. COVID-19 caused only a marginal decrease in visits per month (*P*=.08) at AR1, whereas the site without an established program (AR hospital 2) had a 3-4 week gap in care while the program was initiated. Cases at the LTAC hospital tended to be more complex and difficult to manage virtually. The telehealth usability questionnaire median category scores were 7 for *Ease of Use*, 8 for *Interface Quality*, 6 for *Reliability*, and 9 for *Satisfaction and Future Use*.

**Conclusions:**

The virtual vision clinic process improved inpatient access to eye and visual neurorehabilitation assessment before and during the COVID-19 quarantine and was well accepted by providers and patients.

Vision problems are common in inpatient rehabilitation facility (IRF) stroke and brain injury units, affecting 60%-70% of patients.^1^, ^2^, ^3^, ^4^, ^5^ Strabismus (misaligned eyes) occurs in approximately 1 in 5 survivors of stroke, causing functional impairment, reading difficulties, and mobility challenges in elderly persons, with 2.2 times increased odds of falls with musculoskeletal injury^6^ and substantially decreased quality of life scores.^7^ Homonymous visual field defects are also very common, occurring in 29%-50% of survivors of stroke,^2^^,^^8^ causing reduced detection and delayed responses for obstacles when walking^9^, ^10^, ^11^ and driving,^12^^,^^13^ affecting independence^11^ and quality of life,^11^^,^^14^ and likely increasing risk for fall, readmissions, and barriers to community re-entry.

Neurologic visual impairments might be addressed during the inpatient stay with vision rehabilitation. Vision rehabilitation has been defined as the process of treatment and education that helps individuals who are visually disabled attain maximum function, a sense of well-being, a personally satisfying level of independence, and optimum quality of life.^15^ Visual neurorehabilitation is a subspecialty in this field^15^ involving a multidisciplinary team that may include Doctors of Optometry (ODs) (residency-trained in low vision, neuro-optometry, or both), occupational therapists (OTs) specializing in visual/perceptual deficits or low vision, or orthoptists. In the United States, ophthalmologists only rarely specialize in vision rehabilitation; however, the American Academy of Ophthalmology advocates for vision rehabilitation.^16^ Neuro-ophthalmologists may staff IRFs or act as external consultants, having a higher level of expertise in diagnosis and medical management but with less emphasis on prism and rehabilitation strategies than ODs. OTs have a significant role in addressing visual function issues at IRFs, and some may have postgraduate certification in low-vision rehabilitation through the American Occupational Therapy Association.

The visual neuro-rehabilitation process may include but is not limited to visual diagnosis, education, compensatory training, restorative therapies, assistive technology, and ophthalmic prism application. Fresnel press-on prisms are frequently applied by ODs and ophthalmologists for strabismus to restore binocular vision and sometimes for homonymous field defects to expand the visual field and are a valuable part of our preferred vision rehabilitation approach; therefore, the ability to fit prisms virtually was an important consideration in this study. They are inexpensive, can be applied at the time of examination with only a pair of scissors, and can be easily removed or changed as the patient recovers so long as the patient can access an eye care specialist (OD or ophthalmologist). Press-on prism success rates are relatively high with reported ranges of 64%^17^-80%^18^ for strabismus and ∼50% for hemianopia.^10^ The Peli prism design for hemianopia is supported by a double-blind multicenter randomized controlled trial that found significant improvements in self-reported mobility over a sham.^10^ In addition to prisms, compensatory methods may be taught by OTs or ODs, such as positioning the head or reading material in such a way as to reduce double vision in strabismus or by strategically positioning the eyes (eccentric viewing) or frequently scanning toward the blind side in hemianopia.^19^^,^^20^ Oculomotor therapies that aim to restore normal function by asking patients to repeatedly make eye movements in the direction of a weakened extraocular muscle may be used, although the evidence base in neurologic visual disorders is limited to small studies. Our vision rehabilitation protocols (supplemental table S1, available online only at http://www.archives-pmr.org/) contain activities approved by a vision special interest group with representatives from each site (acute rehabilitation [AR] hospital 1, AR hospital 2, and long-term acute care [LTAC] hospital) (table 1). The protocols have been in place for ∼8 years and were not developed for the purpose of this telemedicine program. Aside from prism fitting and initial education, little to no vision rehabilitation training or therapy was provided via the video conferencing platform. The treating OT provided this care in-person after the assessment, reinforcing the education pieces and rehabilitation protocol activities prescribed.

**Table 1:** Vision clinic telemedicine population characteristics

Location	Telecases	Age (y), mean ± SD	Female (%)
AR1	215	71±17	46
AR2	18	61±20	50
LTAC	10	53±19	70

Abbreviations: AR1, acute rehabilitation hospital 1; AR2, acute rehabilitation hospital 2; LTAC, long-term acute care hospital.

Given the negative functional and psychological effects caused by neurologic visual impairments and the availability of inexpensive and effective evidence-based interventions, which can be modified as the patient recovers, beginning the process of vision rehabilitation alongside inpatient occupational, physical, and speech-language rehabilitation is logical. Ideally, an OT and OD would deliver this care as a team; however, in the United States, rehabilitation facilities have limited access to ODs specializing in vision rehabilitation. The reason for this is not particularly well described, but in our experience OD availability to come to an IRF is usually limited to 1-day per week or less, with access to neuro-ophthalmologists being even more limited. While OTs with vision rehabilitation training and experience are routinely employed full-time by IRFs, it is our experience that most prefer to collaborate with an OD or ophthalmologist to conduct the visual neuro-rehabilitation assessment in the interest of providing the highest quality care and avoid scope of care issues. For example, application, training, and fitting of assistive and prosthetic devices is part of the OT scope and may interpreted to include prism and magnifiers for visual impairments^21^; however, OD vision rehabilitation specialists have direct training and experience that is useful to guide the specialized OT (supplemental table S2, available online only at http://www.archives-pmr.org/). Likewise, an OT will typically intervene for symptoms of a homonymous field cut during functional mobility, activities of daily living, and instrumental activities of daily living training by providing multimodal cuing to encourage/facilitate the patient to scan and shift their gaze (clearly within the scope of the OT); however, they often avoid specific eye exercises or dedicated scanning exercises aimed at improving saccadic eye movements (questionable as within OT scope)^21^ despite their known efficacy),^22^^,^^23^ with which an OD can assist. Therefore, a multidisciplinary approach that involves a vision rehabilitation OD and OT is preferable.

The limited access to inpatient eye and vision rehabilitation care can be further compromised during special circumstances, such as the ongoing (at the time of this study) coronavirus disease 2019 (COVID-19) quarantine, during which in-person optometry and vision rehabilitation clinics were suspended. Telemedicine is one possible solution to improve access to inpatient vision rehabilitation assessment and to continue to provide care during the COVID-19 crisis. Barriers to widespread telemedicine use prior to the COVID-19 pandemic were 2-fold. The first was provider liability concerns regarding inadvertent Health Insurance Portability and Accountability Act of 1996 violations that may occur using one of the many remote communication technologies. In February of 2020, the Office for Civil Rights at the Department of Human and Health Services encouraged telemedicine visits and decreed that in the nationwide state of emergency providers would not be held accountable for Health Insurance Portability and Accountability Act of 1996 noncompliance incidents when using telemedicine in good faith.^24^ Second, prior to COVID-19, insurance coverage for telemedicine was almost nonexistent. During the COVID-19 pandemic, Medicare granted payments for telemedicine visits^25^ and many private insurers followed.

To address access to care issues, it may be feasible for the IRF OT staff to administer an application (app)-based visual test battery, operate the teleconferencing equipment, apply press-on prisms under the ODs remote guidance, and facilitate use while monitoring response. While IRF OTs are typically trained to conduct a vision screening, we expected the OD would require additional history and testing along with test reliability parameters to confidently diagnose and recommend appropriate treatment and follow-up. This might be best accomplished with a vision testing app suite with guided history, auto testing distance measurement, fixation monitoring, and adherence to eye covering protocols. The app should provide effective instructions to the OT and patients and produce a report that can be rapidly and securely transferred to the off-site OD and uploaded to the medical record. At the time of this project there were several vision testing software apps available on the app store that might have been combined to create a suite for IRF vision virtual visit examination. A clinical telemedicine product called EyeCare Livea was available, offering video conferencing and an integrated visual acuity testing app; however, no other visual testing functions were available at that time. A tablet-based vision testing approach for stroke had been reported by Quinn et. al., referred to as the StrokeVision app,^26^ but was not available as a clinical product. Prior to this present study, as part of their regular in-person examinations the IRF vision service ODs were using Visual Acuity XLb and occasionally Pocket Eye Examc for iOS, which included acuity, color vision testing, optokinetic nystagmus strips, red desaturation stimulus, and flashlight stimulus for pupil testing. Unfortunately these apps had serious limitations, making them, in our opinion, poorly suited for virtual vision rehabilitation examinations. The former was difficult to learn because of insufficient instructions for the novice user and did not monitor testing distance and therefore could not verify accuracy of testing method. The latter had inaccurate acuity measurement, test results were not recorded, instructions were not given, and there was no visual field testing app. The OD physicians had also been using EyeTurn,d an app for strabismus measurement, which was developed by Massachusetts Eye and Ear and EyeNexo LLC with funding from the National Institutes of Health. Our hospitals were a site for the validation study, which found similar accuracy between the app and standard clinical tools^27^ and was fairly effective to deliver IRF strabismus consult using a store-and-forward approach.^28^ However, the EyeTurn app was narrow in focus: it just measured strabismus angle, and its limitations were that the consult did not provide ocular motility examination such that common strabismus patterns (third, fourth, and sixth nerve palsies) could not be identified, visual acuity was unknown (asymmetrical acuity would preclude successful treatment), history provided was inconsistent, and visual field defects and other visual and ocular issues could not be addressed.

Given the limitations of the various existing mobile apps, a vision testing app suite specialized for visual neurorehabilitation was developed through an engineer-clinician feedback loop, combined with a video conferencing process, and piloted for 1 year as a clinical service at a 60-bed inpatient acute rehabilitation hospital (Spaulding Cape Cod [AR hospital 1], East Sandwich MA), which was retrospectively reviewed.

We hypothesized that the process was well accepted by practitioners and patients evidenced by (1) greater numbers of patient examinations relative to preimplementation, (2) increasing frequency of clinic days from twice monthly to weekly, (3) consistent or increasing utilization over the pilot period, and (4) low complaints/adverse events. We also hypothesized that the telemedicine program allowed continued access to vision rehabilitation assessment during the COVID-19 quarantine when all in-person optometry and vision rehabilitation services were suspended (as were other consultant services). Subsequent expansion of the telemedicine service to a 150-bed acute rehabilitation facility (AR hospital 2, Charlestown MA) and a 180-bed LTAC facility (Cambridge MA) is also described.

## Methods

### Study design and setting

This was a retrospective study of a clinical telemedicine pilot program at an acute IRF, AR hospital 1. All activities described were performed as part of a clinical process, which was later studied retrospectively via record review to determine if access to care was improved. As such informed consent was waived. The clinical process is described along with the retrospective research methods in sufficient detail to allow replication.

The study was conducted in accordance with the tenets of the Declaration of Helsinki. The protocol was approved by the institutional review board at Mass General Brigham Healthcare.

#### Prototyping of an iPad-based visual neurorehabilitation testing suite

**Fig 1:**
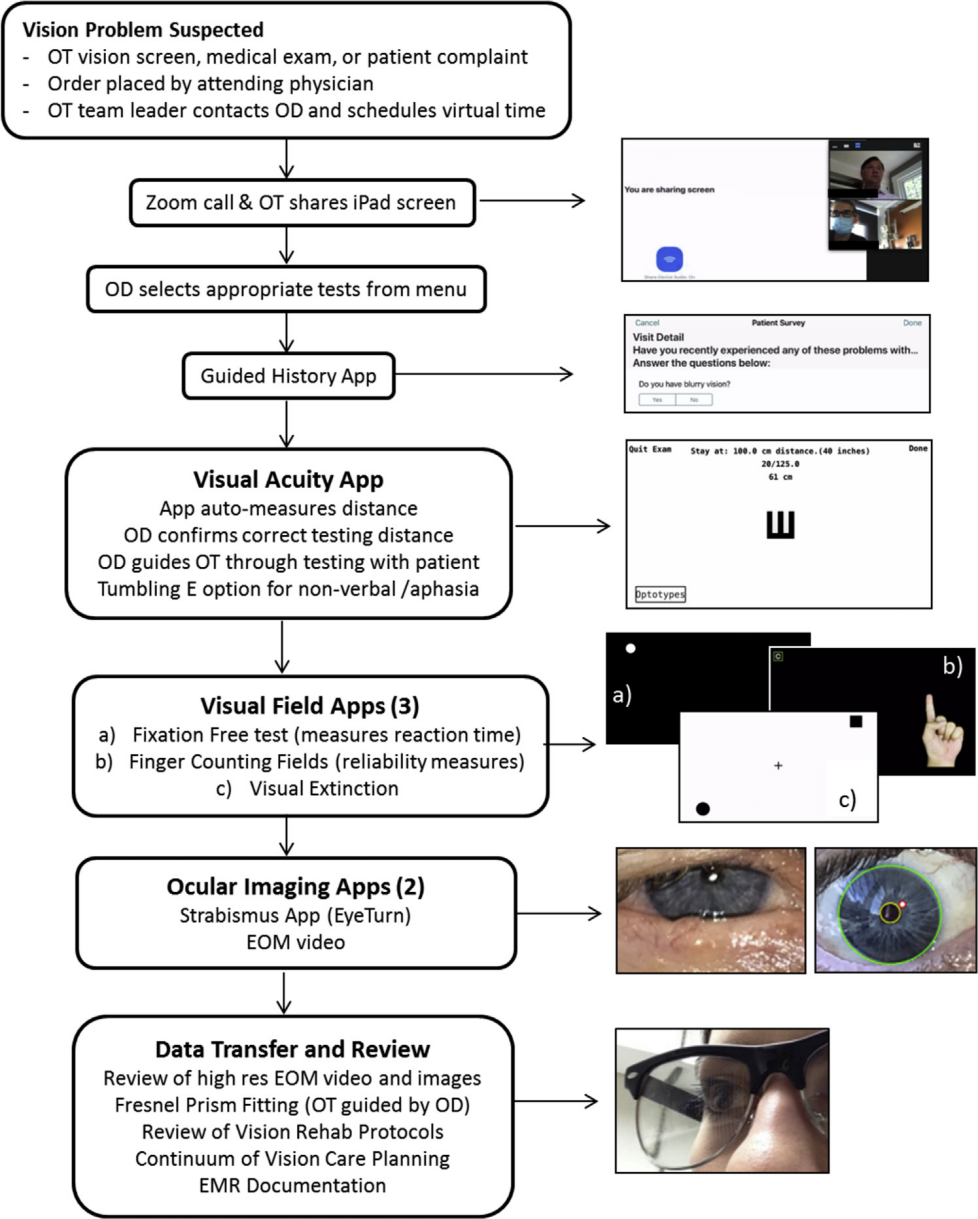
Pilot program clinical workflow for virtual inpatient vision rehabilitation consults using custom iPad vision testing software combined with video conferencing. Note that Zoom with screen sharing was used only at AR hospital 2, whereas AR hospital 1 and LTAC hospital used Partners Virtual Visit software, which did not have screen sharing capabilities. Abbreviation: EMR, electronic medical record.

An existing suite of apps for general eye clinic (EyeXMe) was modified by engineers at EyeNexo with advice and feedback from the clinicians (ODs and OTs) to provide a guided history specific to neurologic vision problems (referred to as EyeXM Rehab). It allowed visual acuity testing with single-letter and tumbling E optotype^29^ options, which were indicated by the clinicians as being conducive to testing patients with cognitive impairment and aphasia. They also requested 3 visual field tests, an extraocular movement (EOM) test which provided instructional cues to the patient/OT, and the EyeTurn app^27^ for strabismus detection and measurement (fig 1). The history app standardized questioning to include important details such as “is the double vision present when covering an eye, how are the double images positioned, do you wear glasses, and do you require reminders to look to the left or right.” Visual field testing software included a finger counting fields test (image of a hand appeared on the screen), a visual extinction test with single and double simultaneous presentation of stimuli, and a novel fixation-free visual field test. The fixation-free field test could predict hemianopia with 76.5% sensitivity and 78% specificity in the neurorehabilitation population when the reaction time difference between right and left fields was >0.5 s (Luo, 2018, unpublished). A 1-cm round white stimulus was presented on a black background, which the patient was asked to touch, with each successive stimulus presented to the opposite hemifield. The difference in reaction time between the right and left fields provided a measure of the functional effect of homonymous field loss as well as some visual field information for patients who could not perform typical gaze-fixed visual field testing. A distance meter function that used the built-in camera was part of the apps and guided the OT to set the correct distance for the visual acuity test and auto-calculated eccentricity of visual field stimuli. Field testing incorporated reliability measures including fixation errors and false positives (catch trials where no stimulus was presented). Testing for hemispatial neglect was not done via the apps. Instead it was performed by the OT as part of the typical assessment in the form of line bisection test, star cancellation, and/or clock dial drawing as well as by observing behaviors during functional tasks. The OD took this information from the OT and compared it with the known locus of pathology to confirm the diagnosis. For example, if OTs reported left neglect behaviors and the *history and physical* or magnetic resonance imaging reports did not support this diagnosis by localizing the pathology to the right hemisphere, diagnosis of left neglect was withheld, and the OD would discuss with the attending physician or refer for additional assessment with neuropsychology. After testing, a PDF report, high-resolution ocular image, and EOM video file were generated and emailed to the OD.

#### Clinical pilot, step 1: in-person evaluations at AR hospital 1 (October-December 2018)

The beta-versions of the app suite were deployed on an IRF-registered iPad Prof and evaluated in person with patients over the 3 months prior to the telemedicine pilot (October-December 2018). The OT doing the app vision evaluation reviewed prior records including the standard vision assessment done by the primary OT and contacted them to determine if there were impairments with functional tasks that might be related to vision or behaviors suggestive of field cut or hemineglect. Then they entered the room and performed the app testing and then brought the iPad to the OD outside the patient’s room. The OD reviewed the findings, mentally constructed a diagnosis and treatment plan based on the app data, and then entered the room to perform the typical in-person examination. This process allowed the OD to evaluate how well he might be able to diagnose the patient virtually if only the app data were available. Notes were taken on the functioning and workflow of the apps to produce a clinician-engineer feedback loop allowing refinements to occur. Qualitative feedback was documented by the clinicians involved in the prototyping (authors M.T., C.C., R.T., K.H., L.M.). By the end of the prototyping process there were 46 updates, and the suite was improved to the point OT and OD clinicians reported reliable results (ie, app testing matched traditional assessment according to the clinicians’ impression). During this phase the OD trained the OTs to apply press-on prisms under direct supervision.

#### Clinical pilot, step 2: onsite telemedicine vision consult evaluations at AR hospital 1 (December 2018)

Two telemedicine sessions with the OD onsite at the IRF were performed with patients after the initial in-person testing but before the telemedicine pilot. The OTs used the app to evaluate inpatients in their room on the second floor and placed a video call to the OD onsite in a first floor conference room. The IRF telemedicine program manager was present to provide assistance as needed, and the medical director reviewed the results at the end of the session. Prior to initiating off-site telemedicine consults, the clinicians discussed limitations with the medical director, namely the inability to evaluate intraocular posterior segment structures. A plan for these types of cases where the physician staff would do an ophthalmoscope examination with the OD being available by video call was planned.

#### Clinical pilot, step 3: offsite telemedicine vision consults (January-December 2019)

The OTs used the EyeXM suite to capture the examination data with patients and uploaded results including a PDF report, EOM video (1280×720 at 10 frames/s), and high-resolution image of the eyes (2823×1210) to a secure file transfer folder,g which was approved by the institution information security department. The OD reviewed the test results and patient medical record and then met with the patient and OT via Partners Healthcare Virtual Visit telemedicine system. During the video call, examination results were clarified and testing could be repeated as needed.

#### Clinical pilot, step 4: expansion of telemedicine vision consult services to other IRF network locations during the COVID-19 crisis (March-June 2020)

To limit risk to the patient and preserve personal protective equipment, in-person optometry and vision rehabilitation clinics were suspended starting March 20, 2020, with a projected return of June 14, 2020. To continue providing vision rehabilitation care, 2 additional sites implemented the described telemedicine process with the following pertinent differences: (1) Zoom Enterprise Editionh was used instead of the hospital virtual visit and (2) training of OT staff was done virtually. Whereas the AR hospital 1 pilot site allowed 30-minutes for the virtual visit, the new sites required 1 hour to account for training and technical challenges. One of the new sites was a 150-bed AR facility similar to the pilot site (Spaulding Boston [AR hospital 2], Charlestown MA), and the other was a 180-bed LTAC facility (Spaulding Hospital Cambridge, Cambridge MA), which was reorganized in April 2020 to care for patients with severe acute respiratory syndrome coronavirus 2 (SARS-CoV-2) requiring a ventilator.

### Research methods

#### Retrospective record review

The billing database at Massachusetts Eye and Ear was queried by a billing specialist for all vision clinic encounters from 2014 to present (June 2020) to obtain counts of patients seen in the vision service as well as demographics and diagnoses. Some patients had multiple encounters that were filtered by an author not involved in clinical care or engineering, using patient identifier and date of service to find and eliminate any duplicate counts. Seven extreme outlier months were excluded including January of 2015, which had an improbable number of examinations (40), included dates where there was no clinic (suspected labeling error in the billing database, which was limited to this 1 month), and included a 6-month period from April to September 2016 when the hospital had just migrated to a new electronic medical record system and only 2 vision examinations were billed over that time. The analysis was performed both with and without these extreme outliers without any difference in interpretation. Results provided are without the outliers because this was felt to be more accurate. The remainder of the data were verified by confirming that the records matched known clinic dates (ie, clinics were nearly always on Tuesdays). Because telemedicine vision consults were not billed until reimbursement restrictions were relaxed amid the COVID-19 pandemic, all telemedicine vision consult encounters (2019-June 2020) could not be tallied from billing data and had to be manually counted from the electronic medical record, which was performed by one of the authors (K.H.). During this manual counting process individual patient records were reviewed in detail for diagnoses; prism response (where applicable); presence of a continuum of care plan; and evidence of any complaints, adverse events, or misdiagnoses. For patients seen in 2020, including all those seen during the COVID-19 quarantine, all records were reviewed. For the other telemedicine vision consults done in 2019, full record review of the entire population was not feasible, and instead a 25% simple random sample was taken.

#### Primary outcome measure

The primary purpose of the telemedicine vision consult program was to increase access to vision rehabilitation care and to determine the effect on access to care during the COVID-19 crisis; therefore, the primary outcome was number of vision consults per month. The hypothesis was that the program would significantly increase the number of examinations and maintain equivalent access to care during the COVID-19 quarantine.

#### Secondary outcomes

Secondary outcomes included number of clinic days per month, utilization rate of the service, refusals, misdiagnoses, and adverse events. Adverse events were reported at the time of occurrence by the onsite OT, by email to the OD. Email inbox search was used to count adverse events, and the OTs were asked to confirm the counts were complete during manuscript preparation. Additionally, providers involved in the teleconsult process were anonymously surveyed using a Likert-type scale with items from the telehealth usability questionnaire, a validated instrument for assessing the implementation of new virtual visit care technologies.^30^ Items that were not relevant to the present study were dropped and wording was slightly modified for context. Components of qualitative information from the OD and 2 OTs involved in the prototyping at AR hospital 1 are also reported (authors M.K., C.C., K.H.).

### Statistical methods

Statistical analyses were performed with Stata/IC 14i or R softwarej; *P*≤.05 was taken to indicate statistical significance. For comparisons against data during the COVID crisis, marginal values .05<*P*≤.10 are also noted, given the relatively small number of cases. The main analysis used a 1-way analysis of variance, with post hoc pairwise analyses as indicated with Bonferroni correction for multiple comparisons to compare the monthly number of vision consults performed before the telemedicine program (2014-2018) during the 1-year pilot in 2019 and during COVID-19 shutdown January through May 2020. Statistical support was provided by the Massachusetts General Hospital biostatistical consulting service, and the author performing the primary analysis (L.M.) was masked to group.

## Results

### Results 1: primary research question: did the number of patients per month who received vision rehabilitation examinations at AR1 increase with the introduction of telemedicine?

**Fig 2:**
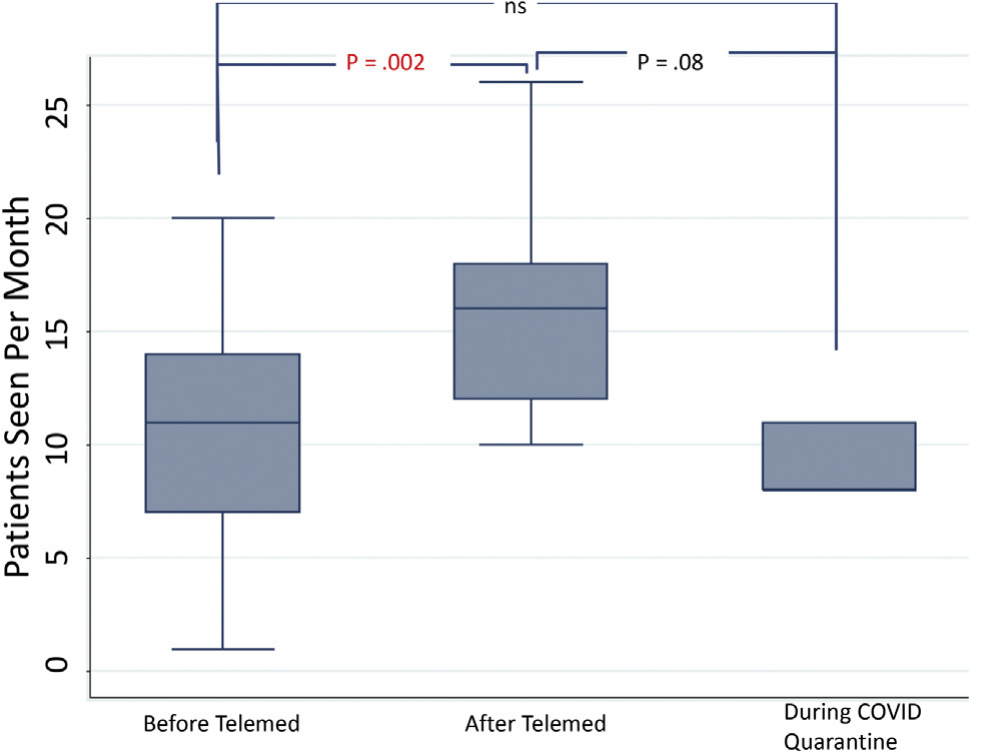
Comparison of no. of patients seen at AR hospital 1 before the telemedicine program (2014-2018), during the 2019 pilot, and during the COVID quarantine (3/2020-6/2020). Boxes represent the 25th-75th IQR, central line is the median, and the whiskers are the ranges. Vision rehabilitation clinic frequency improved from monthly to weekly and significantly increased the no. of patients receiving services. Despite suspension of the in-person inpatient vision rehabilitation service during the COVID-19 quarantine, access to care was not significantly affected, although it was trending toward lower nos. seen, *P*=.08 (Bonferroni corrected).

Data were available for 45 months pretelemedicine from October 17, 2014, to November 27, 2018, and 17 months with telemedicine from January 2019 to June 2020. There was a significant difference between groups, which resulted from an increase in visits per month with telemedicine (analysis of variance F2,59=6.84, *P*=.002). Mean visits per month significantly increased by 4.2 visits per month (*P*=.002), from 10.7±5 pretelemedicine to 14.9±5 with telemedicine (fig 2). COVID-19 caused only a marginal decrease in visits per month (*P*=.08) (see fig 2).

**Fig 3:**
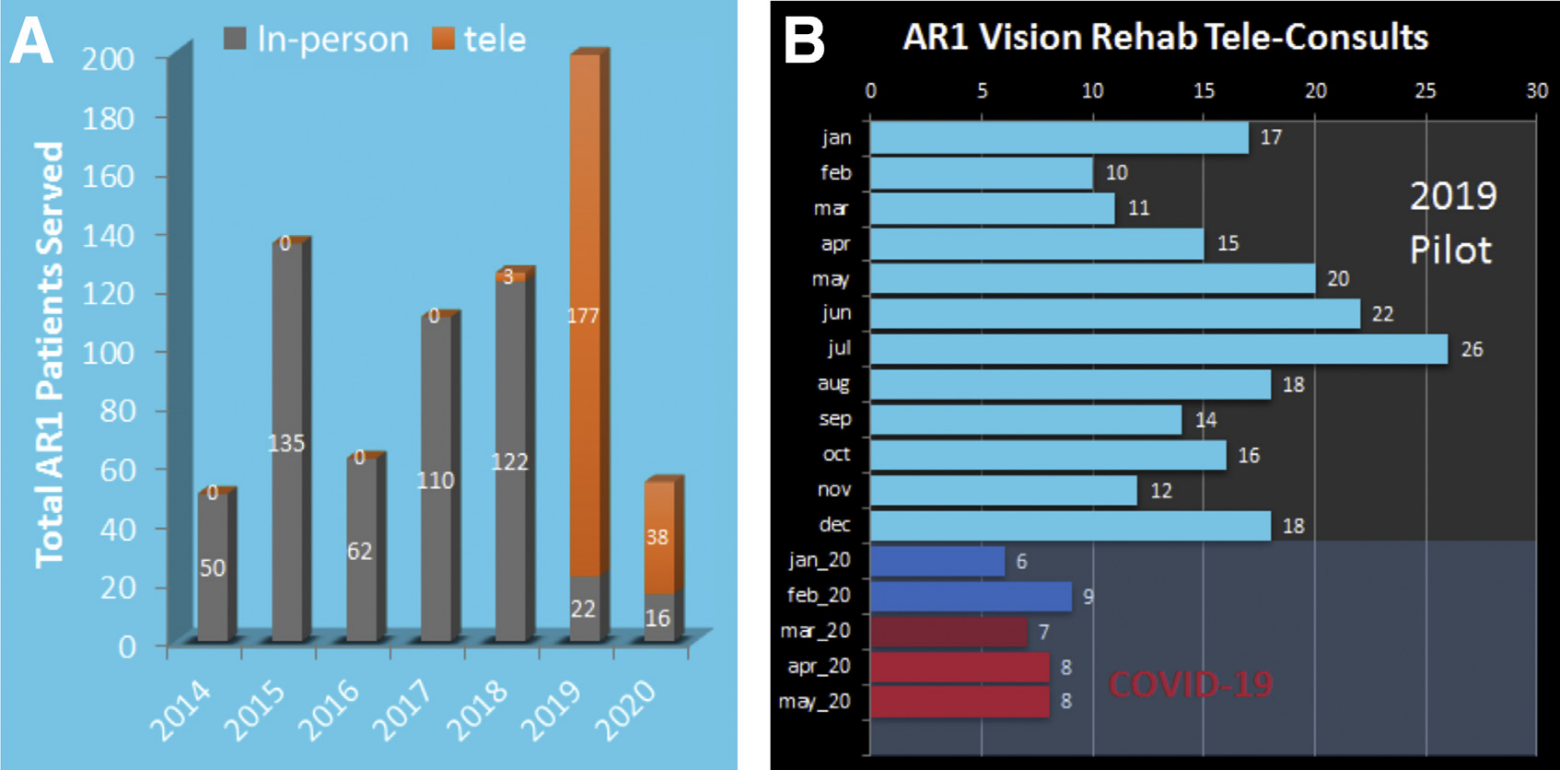
(A) No. of patients served by the AR1 inpatient vision service showed a clear increase in 2019 with the implementation of the teleconsultation program. Orange stack bars are teleconsults. (B) Utilization of the vision rehabilitation telemedicine service was quite variable by month during the 2019 pilot (light blue bars) but never dropped below 10. Values represent total patients seen, not averages. During the COVID-19 quarantine (red bars), access to vision rehabilitation care continued but at a marginally lower rate. Abbreviation: AR1, acute rehabilitation hospital 1.

There was also a significant increase in the number of clinic days per month, from a mean of 2.1±0.8 before telemedicine to 3.5±0.7 with telemedicine (*P*<.001). Figure 3A shows a year by year breakdown of the total patients served before and with the telemedicine program. As can be seen, the number of patients served in 2019 was higher than previous years, and the majority were involved with telemedicine vision consults. Utilization of the vision rehabilitation telemedicine service at AR hospital 1 over the pilot period was consistent, never dropping below 10 patients in a month during the 2019 pilot (fig 3B).

### Results 2: adverse event, complaints, and refusals at AR hospital 1

One patient scheduled for telemedicine vision consult started the process and then refused to continue, citing fatigue and discomfort. Another was unable to do most tests and specifically could not tolerate the flash used in the computerized Hirschberg strabismus test (EyeTurn app), and so it was recorded as a mild adverse event. Qualitative reports suggested there were at least several other cases where patients could not participate in the examination because of impaired cognition, aphasia, or low arousal. Some patients had difficulty tolerating the flash during imaging, particularly when repeat testing was needed. There were no formal or incidental complaints by family members, caregivers, medical staff, or other therapists. No cases of misdiagnosis were identified in the record reviews or by professional communication. A typical time for the entire process in the later stages of the pilot was about 35 minutes, which is very similar to in-person consultation.

### Results 3: expansion of vision clinic telemedicine program to an LTAC hospital

The vision clinic telemedicine program was implemented at the 180-bed LTAC hospital in June of 2019. Ten evaluations were performed over that period through May 2020 (11mo). The population had a mean age of 53±19 years, was 70% female, and had medical diagnoses of brain tumor (2), stroke (4), and 1 each of aneurysm, traumatic brain injury, hypoxic brain injury, and other. Visual diagnoses included strabismus (5), hemianopia (5), hemineglect (2), floaters (1), and blurred vision (1) (note that 5 patients had multiple visual diagnoses). One patient with strabismus, hemianopia, and hemineglect was found to be blind in 1 eye related to the history of present illness. The condition was later found by in-person dilated fundus examination to be Terson syndrome related to her subarachnoid hemorrhage, treatable by vitrectomy. During the COVID-19 crisis, 2 patients at the LTAC were seen for vision rehabilitation issues unrelated to COVID-19, 1 of whom had previously been intubated for SARS-CoV-2 but not at the time of examination. An order was written for 1 patient on the COVID vent unit for red eye symptoms but could not be seen because of infection control policies that prevented bringing the iPad into the room.

### Results 4: expansion of vision clinic telemedicine consults to AR hospital 2

**Fig 4:**
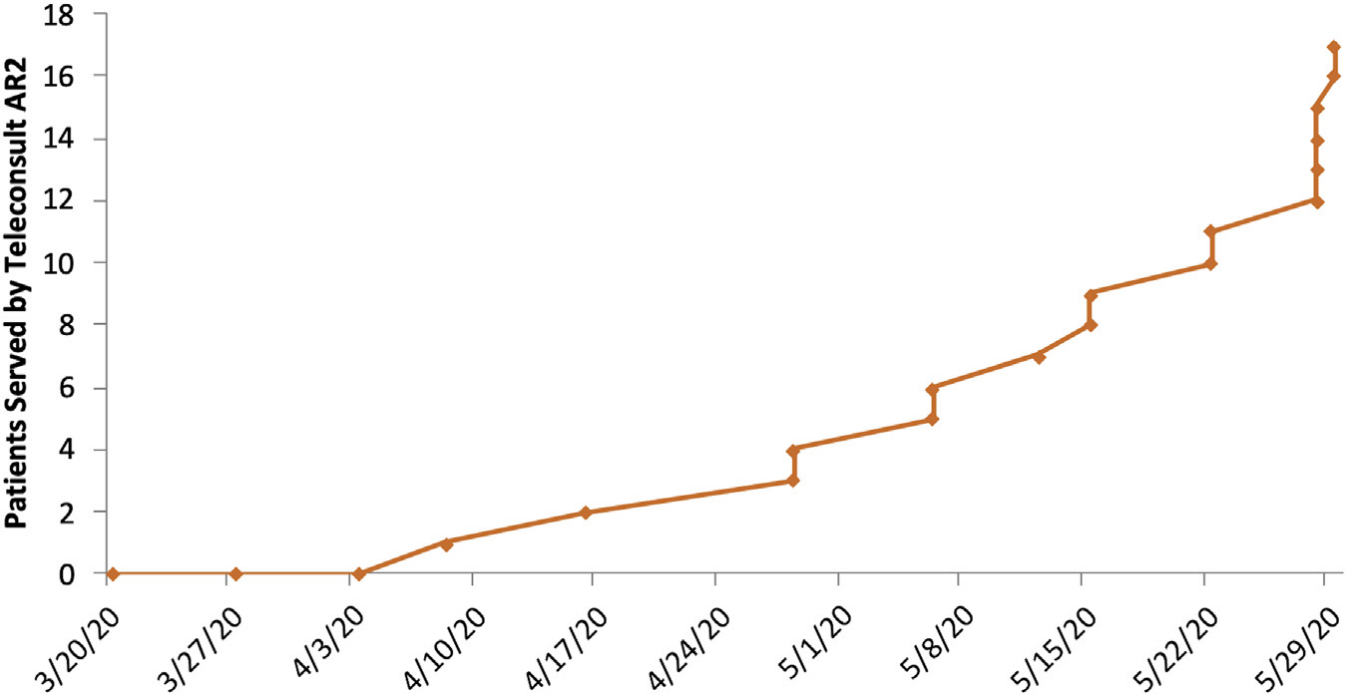
The rate of teleconsults increased exponentially at the new AR2 site during the COVID-19 quarantine, suggesting successful implementation. Abbreviation: AR2, acute rehabilitation hospital 2.

In response to COVID-19, the comprehensive vision clinic telemedicine service was quickly implemented at an urban 150-bed AR facility (AR hospital 2), with 18 patients being evaluated virtually during that 2.5-month period. The cumulative patients served by telemedicine grew exponentially over that time (fig 4). The population had a mean age of 61±20 years, was 50% female, and had medical diagnoses of stroke (6), traumatic brain injury (2), brain tumor (2), respiratory distress (3), and other (5). Three had been COVID+ in the months prior and had recovered by the time they were examined, with visual diagnoses of blurred vision/dizziness, interstitial keratitis, and homonymous hemianopia. The patient with interstitial keratitis was sent to a cornea specialist, who indicated it was likely related to the prior COVID-19 infection. The AR hospital 2 population also changed somewhat during the COVID crisis. The hospital accepted more patients with COVID-19 who needed postacute care rehabilitation, and the interdisciplinary team worked together to manage the complications of COVID-19, including acute cerebrovascular disease, deconditioning, and critical illness–associated weakness.^31^^,^^32^ To accommodate the potential increase in patient load, each floor typically designated for a specific patient population (eg, traumatic brain injury, stroke, spinal cord injury, amputation) started to provide beds for those with a different admission diagnoses.

### Results 5: aggregate telemedicine data

**Fig 5:**
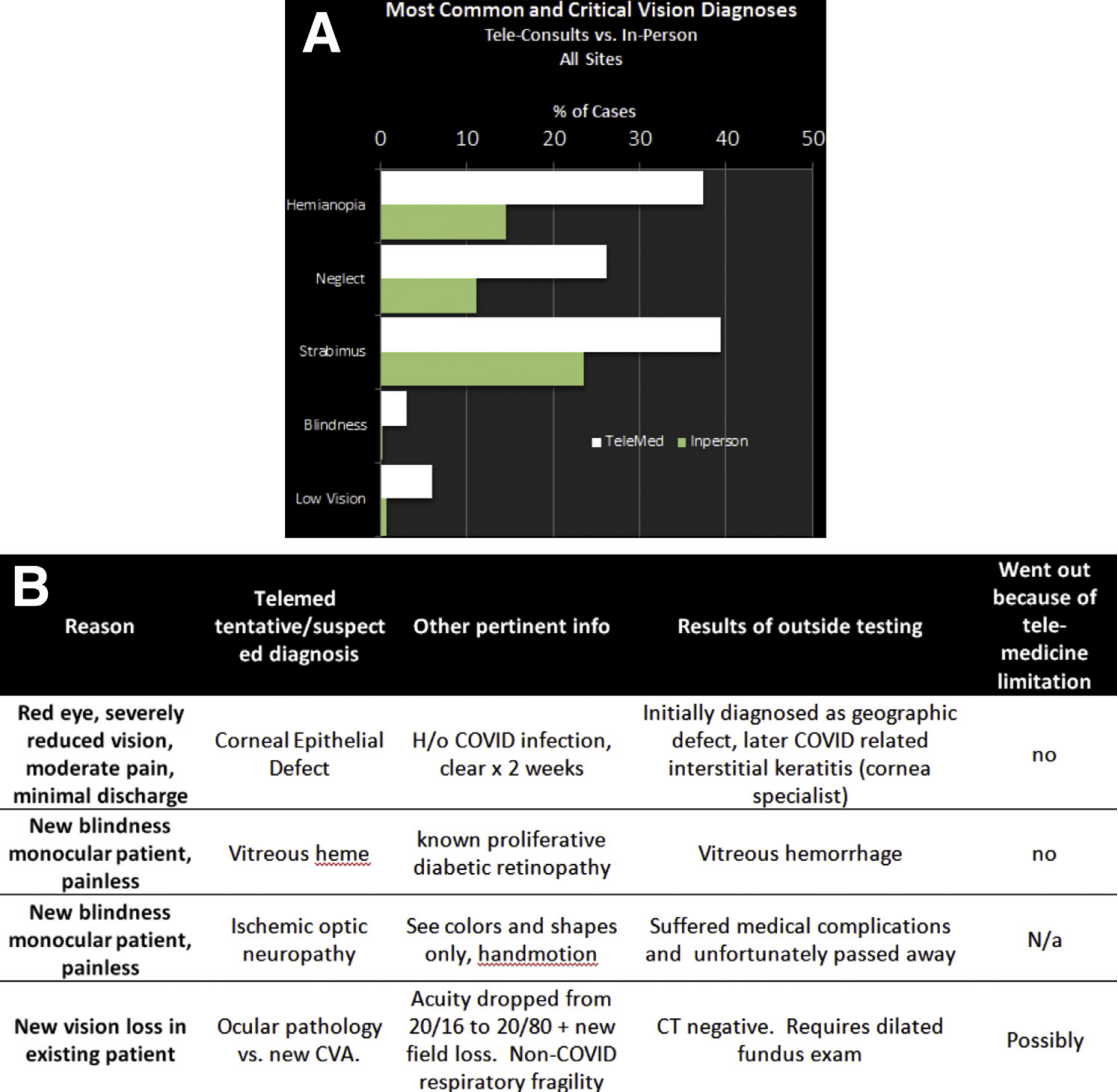
(A) Incidence of common and critical visual diagnoses in patients seen by teleconsult (white bars) compared with in-person visits. A greater proportion of hemianopia, neglect, and strabismus in telemedicine may reflect the suitability of the teleconsult platform to address these issues. (B) Four patients seen by teleconsult had to be sent out for urgent evaluation, which may have been in part because of an inability of the technology to evaluate pathology of the ocular posterior segment. Abbreviations: CVA, cerebrovascular accident; CT, computed tomography; H/o, history of; NA, not applicable.

In total across all sites and years, 237 telemedicine vision consults had been performed, 99 of which were reviewed in detail, including all 38 cases from 2020 and a 25% random sampling from 2019 (see Methods). The most common visual diagnoses seen virtually were strabismus (39%), hemianopia (37%), and neglect (26%) (fig 5A). Note that some patients had multiple diagnoses so totals do not sum to 100%. Prism was tried by the OT with OD virtual oversight in 40% of cases (40/99) and was accepted, at least initially in a short trial, in 83% (33/40). Further breakdown found that prism was tried in 49% (19/39) of strabismus cases, 46% (17/37) of hemianopia, and 8% (2/26) of neglect (when the patient also had hemianopia or strabismus). Acceptance rates were 94%, 74%, and 100%, respectively. Data on long-term acceptance were not available. Critical visual diagnoses included total or near-total blindness in 3% of cases and low vision in 6%. In-person examination data from AR hospital 1 are provided in fig 5A for comparison, showing a substantially higher proportion of strabismus, neglect, hemianopia, low vision, and blindness in the virtual visits. Four patients seen by telemedicine vision consult at 2 sites (2 each) had to be sent out for urgent evaluation and are described in fig 5B. Likely all of these cases would have needed to be sent out even with in-person care because they were visually threatening and required ophthalmologist intervention.

### Results 6: perceived value and telemedicine usability questionnaire

**Fig 6:**
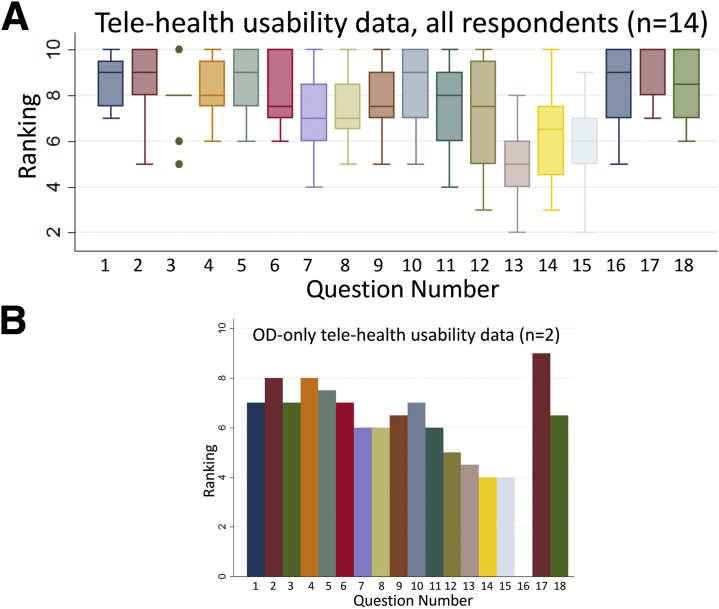
Telehealth usability questionnaire results. Of the 20 providers involved in the teleconsult process, 14 completed the anonymous questionnaire (70%). The box plot data represent the group median and 25th-75th IQR for each question. The whiskers represent the range of data with outliers denoted as dots. Question 3 had 10 responses, 6 of which had a ranking of 8, explaining the lack of an IQR box.

A total of 17 OTs, 2 ODs, and 1 medical doctor (physiatry resident physician) were involved in use of the vision clinic telemedicine consult software, and 14 responded to the questionnaire (70%). The data are presented for all respondents and for just the ODs, who may have had different ratings because they were required to make clinical judgments based on the technology. Visual inspection of the data (fig 6A vs B) showed similar responses to other respondents, a median OD overall satisfaction of 6.5. Ratings for the “same as in-person” question were 5 and 4 for the 2 providers. The overall median (interquartile range [IQR]) category scores were 7 (IQR, 7-7) for *Ease of Use*, 8 (IQR, 8-8.75) for *Interface Quality*, 6 (IQR, 5-6) for *Reliability*, and 9 (IQR, 8-10) for *Satisfaction and Future Use*.

## Discussion

This study investigated the use of a new telemedicine service consisting of a suite of custom iPad vision testing apps and a video conferencing system that intended to improve access to inpatient visual rehabilitation care before and during the COVID-19 pandemic. At the time of this report, 237 virtual visits had been provided across 3 different inpatient sites including 2 acute IRFs and 1 LTAC hospital.

The main finding was a significant increase in the number of patients who received vision rehabilitation consultation during the 1-year pilot. The increase is best explained as being a direct result of the new telemedicine service. The frequency of vision clinics during this period increased from twice monthly to weekly, which given the average length of stay of ∼2 weeks allowed access to more patients who needed care. Prior to the telemedicine service, patients who were admitted on or immediately after a vision clinic day were discharged before they could be seen; an issue that was successfully addressed with telemedicine. The virtual vision clinic service was consistently utilized during the 1-year pilot period, with no fewer than 10 consults per month and as many as 26 (see fig 3B), reflecting both the need for the service and indirectly supporting its efficacy. Overall patients seemed pleased with the process and only 1 refused care via telemedicine consultation after starting the examination process. The whole process took 30-45 minutes per patient at the more experienced AR hospital 1 site and 1 hour at the other sites. Hospitals implementing a similar process may want to schedule an hour initially until staff are more experienced. It was also helpful for OTs new to the process to have the OD on the video call during the app testing to guide the process; however, once proficient it may be feasible to have the OD join only after the testing is complete. In our clinics OTs were always involved in the vision testing, contributing their specialized training and experience to the process to ensure examination data were as accurate and reliable as possible. This is the preferred approach as opposed to training a technician or OT aide to perform the testing.

Data from the validated telehealth usability questionnaire was obtained anonymously and should therefore represent a valid opinion of the process. Findings were encouraging with a median *Overall Satisfaction* score of 8 of 10 (see fig 6, question 18). The lowest scores were in the reliability category, suggesting the users did not think the service was the same as in-person care and that it was difficult to recover in the software after making an error. This is likely because of the iPad app suite having been originally designed for waiting room patient self-administration and so was specifically written to prevent the patient from going back to repeat a test. This known issue could have been addressed but required a major rewriting of the code, which was not feasible at the time. Fortunately this did not prevent the process from being successful and can be interpreted as more of an inconvenience. If retesting was needed or a test was accidentally skipped, the software allowed the user to start a new session and select just the necessary tests, requiring only a couple minutes of additional time. Improving the “similarity ranking” between virtual and in-person care would likely require development of methods for posterior pole examination, pupil measurement, intraocular pressure measurement, enhanced ocular surface evaluation, and improved video call interface.

There were no complaints or misdiagnoses reported or found by record review. The record review further indicated high short-term acceptance rates of prismatic interventions prescribed over telemedicine. The ODs reported being comfortable enough with the examination data to make key diagnoses related to the specific problem-focused consult request and implement vision rehabilitation. The ODs had access to neuro-ophthalmology consult when needed within the health care network, although it required transfer to an outside facility. In any case the ODs worked within their training and scope of practice to make diagnoses to the level possible in the telemedicine format, planning for in-person evaluation when possible. In many cases the OD and physiatry continuum of care plans included an evaluation with neuro-ophthalmology after discharge. While not available at the time of this study, it may be possible to receive problem focused neuro-ophthalmology e-consult using the same app examination data.

Inpatient visual neuro-rehabilitation may be among the best early use cases for telemedicine in eye care. The majority of vision conditions encountered in the IRF, shown in fig 5A, lend themselves well to subjective visual testing or external imaging with little need to immediately visualize the inside of the eye or perform biomicroscope examination of the cornea. Patients are in an environment where they have the support of skilled OTs who can assist with setting up and operating the technology and collecting reliable examination information despite the challenging patient population. After the consult the OT and physiatry staff can monitor for changes in visual symptoms and reinforce strategies and therapies to deal with the vision impairment. The data here also suggest it is quite feasible for the OT to apply prisms under the virtual supervision of the OD, with an 83% initial fitting success rate (see Results section). Prism during a virtual visit was only utilized by 1 of the ODs for the reported period, and so it is unknown what the variation in success rate may be between providers. An OD specializing in low-vision rehabilitation or binocular vision with expertise in fitting Fresnel prism for strabismus and hemianopia (Peli lens) and an OT with specialty in visual perceptual deficits are recommended to maximize the likelihood of achieving similar success rates. Applying press-on prism for strabismus during the early recovery period could be problematic if it were not feasible to obtain timely follow-up to monitor for changes. Our approach, in-person or via telemedicine consult, was to undercorrect as much as possible while still providing symptom relief. With this approach the patient “healed into the lens correction,” becoming even better over the course of a few weeks. Follow-up was generally performed within 2 months from fitting, preferably in person but also virtually during the shutdown.

### Expansion to the LTAC hospital

LTAC hospitals present unique challenges for vision care because patients are often very ill, have extremely complicated medical histories, and are often not able to participate in traditional vision examination. The need for eye dilation and other tests that are not possible with this telemedicine technology is proportionally higher at the LTAC hospital. This particular LTAC site is home to the network’s disorders of consciousness program, constituting approximately 50% of in-person vision service consults. With an inability to test pupil function, visual startle, or optokinetic response, the value of the current suite of apps was limited. Still, there were 10 patients over the 11 months reviewed who were able to participate in the virtual examination. The app suite was also used by specialist OTs in advance of the onsite vision clinic to reduce the time the OD had to spend with each patient, allowing more patients to be seen in a clinic day. Such a process may be valuable for similar hospitals. For all the sites, it was important to have an onsite OT directing the vision clinic who was familiar with the process and could help select appropriate candidates.

### Coronavirus disease 2019

Results showed that the COVID-19 shutdown, which resulted in suspension of inpatient consultant services across the rehabilitation network, only caused a marginal decrease in the number of patients receiving vision consultation at the established AR hospital 1 site. Implementing the program at AR hospital 2 during the COVID-19 crisis where none existed previously was much more challenging, with a gap in care of about a month (last in-person clinic on 3/13/20 and first virtual visit on 4/8/20). It took an additional month for staff to become comfortable with the process, utilizing it at a rate more consistent with the in-person service (5-10 patients/wk). Staff also attempted to use the virtual vision clinic process for 2 patients with eye problems and a current or recent history of SARS-CoV-2 as well as for a medically fragile patient at high risk for mortality. None of these 3 cases could be managed by telemedicine, and the patients required transfer to an ophthalmic facility. In-person optometry evaluation capable of hand-held slit lamp, tonometry, and dilated fundus examination may have prevented the need for transfer in 2 of these patients. Nevertheless, the physiatry physician (author Y.C.) reported high value of the telemedicine consult to help triage and provide valuable clinical guidance to physicians and patients. Telemedicine visits have increased considerably for all medical disciplines during the COVID pandemic as both providers and patients become more comfortable with the technology and enjoy the convenience of virtual visits. Medicare is considering making the telemedicine expansion that occurred for COVID-19 a permanent change, which would encourage more clinical outcome research comparing telemedicine with in-person visits.^33^ With reimbursements secured, technology improvements and increasingly user-friendly and secure apps will be developed to improve on this platform that is presently in its infancy. As the mobile technologies rapidly evolve, most mobile devices will come with much boosted artificial intelligence computation power, fast wireless connection (eg, 5G), higher frame rate cameras with higher light sensitivity, and displays with higher resolution. We anticipate that these advances will further support virtual eye care, allowing more comprehensive ocular health assessment of the cornea and retina.

### Study strengths, limitations, and future directions

The current study used methodologies to improve scientific rigor, including masking of the author performing the primary statistical analysis, use of an anonymous and validated questionnaire, and the criterion standard simple random sampling to select charts for review. A limitation is the possibility that the increase in vision consults was because of some other factor unrelated to the availability of telemedicine consults, but this is unlikely, and the effect size is fairly large. The lack of an effect of COVID on consult rate (ie, there was not a significant reduction in vision rehabilitation consults) may represent the efficacy of the telemedicine program; however, it may also have failed to reach significance because of being underpowered with a relatively small sample size. Fortunately there were not more months of the shutdown by which to evaluate this. This finding should therefore be interpreted with caution in the sense that if other IRFs plan to implement a similar program (eg, in anticipation of another quarantine in winter of 2020-2021), they may still see a significant drop in vision consults provided. Still, results suggest that access to vision care should be improved, with a similar telemedicine program with low risk for misdiagnoses or other adverse events both during a crisis and as a long-term solution. Future similar studies are needed at other IRFs to evaluate if the approach is successful with different providers in slightly different environments. To more definitively confirm safety of the approach, a study involving a direct comparison between in-person and virtual vision clinics would be helpful. The value of additional examination data provided by the suite of apps compared with an entirely video conference–based examination might also be studied for suspected benefits of using the apps. In our experience, the resolution on the video call was not sufficient to adequately assess the patient, and having high-resolution photos and videos as part of the evaluation was essential.

## Conclusions

Access to vision rehabilitation care was improved using a telemedicine system consisting of a suite of vision testing apps to compliment the OT vision screening combined with a video conference system, evidenced by an increase in the mean number of patients seen per month and increase clinical sessions from twice monthly to weekly. The access was maintained even during the COVID-19 quarantine period. Quality of care did not appear to suffer with this approach, with no complaints or serious adverse events and a prism fitting success rates similar to in-person care.

## Suppliers

a. EyeCare Live; Eyecarelive Inc.
b. Visual Acuity XL; Kybervision.
c. Pocket Eye Exam; NOMAD.
d. EyeTurn; Massachusetts Eye and Ear and EyeNexo LLC.
e. EyeXM; EyeNexo.
f. iPad Pro; Apple Inc.
g. File transfer folder; Dropbox Business.
h. Zoom Enterprise Edition; Zoom.
i. Stata/IC 14; StataCorp.
j. R; R Core Team.
