# Supplementary material for: Inpatient Virtual Vision Clinic Improves Access to Vision Rehabilitation Before and During the COVID-19 Pandemic
Source: Arch Rehabil Res Clin Transl. 2020 Dec 19;3(1):100100. doi: 10.1016/j.arrct.2020.100100 (PMC7749728; doi:10.1016/j.arrct.2020.100100)

PROTOCOL FOR HEMIANOPIA REHABILITATION

1. **Patient Education and Awareness Training:**
   a. Patients with field defects most often are in denial that they fail to detect things, report they miss things only because they have been told so, rarely fully understand that they are blind in one half of each eye, usually think the eye on the side of the defect is blind, and don't understand that the blindspot moves when they move their eye. As a result they will complain that sometimes they can see, and sometimes they can't.
   b. Discuss the cause of vision loss
   c. Personal Space Demo: Patient looks at examiner and holds up both hands (like being sworn into court).
   d. Examiner shows them how they can't see their hand on the blind side unless they look over.
   e. Have them explore the edge of their blindspot with their own hand. EYES MUST REMAIN STATIONARY.
   f. Move from non-seeing to seeing
   g. Repeat moving from seeing to non-seeing
   h. Practice all along the edge of the defect for ~2min.

2. **Eccentric Viewing Training:**
   a. Once they recognize the blind field, demonstrate how it worsens when looking away from it, and improves when looking towards it. Continually ask the patient where the defect is on every visit multiple times.
   b. With the hands still up in the blindspot, have patient look to the wall to the side of the defect.
   c. Keep eyes still, pointed at the wall
   d. Instruct them to pay attention to the rest of the room being careful not to move the eyes.
   e. Ask if they can see most of the room and their hand that was previously in the blindspot in their peripheral vision.
   f. Instruct them to look to the opposite wall.
   g. Ask them to notice how the blindspot was drawn across the room like a shade.
   h. Ask them to notice how they cannot see you directly in front of them. Watch their eyes- they often scan and report they can see everything.

3. **Anchoring and visual search strategies**
   a. General Observation: "How much can you see in a room".
   b. Right hemianopes should try stay to the same side of the room or hallway as the field cut. An immediate and significant improvement in their mobility will be seen. For left hemianopes this is not advised since it forces them to walk against traffic increasing risk of collision.
   c. Give an anchor 3-4 feet from them to continually scan back to (eg. Baseboard).
   d. When crossing threshold, patient should point eyes at door frame on the side of the field cut.
   e. Eventually force the patient to choose their own anchor.
   f. Saccadic Fixator/Dynavision: Place a piece of black tape on the edge of the device on the side of the field cut to act as a visual anchor. Practice returning to the anchor after each light is depressed.

4. **Reading Rehab**
   a. Black or red line page anchor on side of the field cut.
   b. Have patient use finger to guide eyes.
   c. Try line guides: Ruler or folded paper
   d. Trial large print

*Goal is for patient to employ any effective strategies without cueing prior to discharge

P-Prism Demonstration Training

Peripheral prism glasses (p-prisms, aka Peli Lens or EP prisms) provide visual field expansion, easily documented on standard perimetry in patients with homonymous hemianopia (HH) (Peli 1998, Peli 2000). This approach employs monocular 40-57 prism diopter (20-25 degrees) sector prisms that are limited to the peripheral (superior and inferior) lens (Fig. 1. c & d) but span both sides of the pupil, and thus expand the visual field at all lateral positions of gaze. Prisms of 40Δ were used with no difficulties in our first multicenter clinical trial(Bowers, Keeney, Peli 1998).[36] The field expansion provided was found to be effective in helping patients with HH avoid obstacles while walking. Out of 43 patients with HH without SN, about 50% were still wearing the prism glasses after 12 months, reporting improved mobility. While p-prisms expand the accessible visual field immediately on first application, the perceived direction of objects detected through the prism is incorrect; objects on the blind side appear to be on the seeing side. For this reason patients receiving p-prisms must be trained to use them.

**Permanent P-prisms**	**View as seen by a left hemianope with p-prisms. The shaded area is unseen by the patient.**
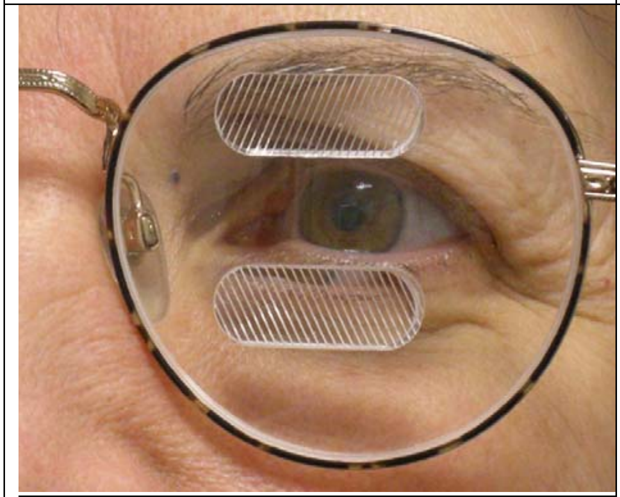	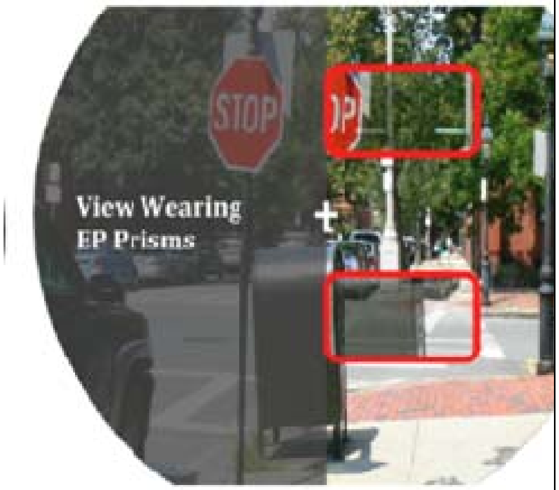

Summary of how the prisms should be used

- In many ways training and adaptation resembles that required of first time bifocal wearers, where head movements are needed to eliminate the blurry appearance of objects seen through the wrong part of the lens.
- Patients should be taught to look through the central prism-free area of the lens at all times.
- The patient should never look into the prisms otherwise they will experience central diplopia (double vision), which is disorienting and uncomfortable.
- The prisms act in peripheral vision. When an object of interest is detected via the prism, the patient should then turn his/her head and eyes to directly view the object with central vision through the central, prism-free, part of the lens.

- Objects detected via the prisms will appear to be shifted towards the seeing side. The patient will have to turn his/her head and eyes towards the blind side in order to view the object with central vision.
- These head and eyes movements require deliberate attention at first and should become almost automatic following training and practice.

Adjusting the Prism Glasses

- Adjust the **vertical** position of the prisms by first pushing the glasses up or down on the nose so the prism-free area is centered on the pupil. Adjust the nose pads as a last resort.

Demonstrate the visual field expansion effect of the prisms

- This procedure is similar to confrontation visual field testing, but is done under binocular viewing conditions (i.e. with **both** of the patient's eyes open).
- Face the patient at eye level from a distance of 20 – 30 inches.
- Ask the patient to look directly at your nose through the center of the lens and NOT THROUGH THE PRISMS.
- With the patient still looking steadily at your nose, now move your hand in from the blind side into an area covered by the (upper) prism. Bring your hand in along a line about 2 inches above the pupil center (the horizontal midline). Ask the patient to report when they can detect your wiggling fingers. Your fingers should first be detected when still in the blind field, about 8 inches (25°) from the vertical midline. Repeat this for the lower field. The patient should be able to detect the movement in their peripheral vision, upper and lower, via the prisms while looking through the center of the lens, NOT THROUGH THE PRISMS.
- Demonstrate to the patient the difference between where the fingers were first detected without, and then with the aid of the prisms, by asking the patient to look directly at the fingers in each case through the prism-free portion of the lens.

Practice turning head and eyes to directly view object on blind side

- With the patient looking steadily at your nose, move your fingers again in the blind field in an area covered by the prisms.
- When the patient detects your moving fingers in the prism tell them to then look directly at your fingers by turning their head and/or eyes towards the blind side to view your fingers through the (prism free) center of the lens.
- Repeat this 60-100 times with your fingers in different positions for the upper and lower prisms.

Demonstrate undesirable central diplopia when patient looks directly through prism

- Ask the patient to look directly through either the top or bottom prism.
- By necessity, he/she will also be looking through the non-prism portion of the other lens. This will cause CENTRAL diplopia. Tell the patient that this is to be avoided.

Reach and touch – real and apparent positions of objects detected via prism

- This exercise will increase the patient's understanding of where objects detected via the prisms really are in relation to where they appear to be.

- With the patient looking at your nose, move your fingers in the blind field in an area covered by the prisms.
- Ask the patient to grab at your finger as soon as he/she detects it through the prism while still looking straight at your nose.
- Chances are that the patient will miss your finger on the first try. He/she will tend to grab at the apparent position of the finger, which is closer to the seeing field than the real position. Explain this to the patient – explain that objects detected in the prism will seem to be closer to the seeing side than they really are.
- Have the patient repeat the exercise 60-100 times for each prism zone.
- If the patient is unable to perform this task within five minutes of practice (during the dispensing of the prism glasses) they will not qualify for the study.

Training walk

- Take the patient for a walk along a hallway and through uncluttered rooms. **The patient should be escorted at all times.**
- Progress to more cluttered rooms such as a reception area with tables and chairs. Finally, take the patient up and down a set of stairs.
- The training walk should ensure that the patient understands how the prisms give an indication of obstacles on the blind side.
- When a patient detects an object on the blind side (e.g. door frame, chairs, tables) from one of the prisms, he/she should be instructed to look directly at the object through the prism-free portion of the lens.
- Ask the patient to walk up and down some stairs. Walking upstairs should present no problem. When walking downstairs the patient should be taught how to avoid looking through the lower prism. Either the head will have to be lowered to see the stairs through the central prism-free area, or the patient will have to look underneath the lower prism. Similar problems are encountered by bifocal wearers and will require an adaptation period. Advise the patient to use handrails whenever possible.

Prism Adaptation Therapy

Indications: Left Hemispatial Neglect with Right Frontal, Temporal, Parietal, and/or Occipital Lobe Injury

Equipment Needed:

- Base Left 17.6PD (10°) Prism Adaptation Glasses

Step 1 Task Set-up and Practice:

- Place two targets (eg. small pieces of tape) 1-2 inches left and right of midline
- Have the patient place their right hand at their midline (navel or sternum) and left hand on their lap
- Instruct the patient to "look at the target and reach quickly to touch it"
- Instruct the patient to return their arm to the starting position at their sternum after each trial
- Practice until they seem to understand the task

Step 2 Adaptation:

- Have patient close eyes and not open until instructed to
- Place the prism glasses on the patient, bases (thick side) to the left.
- Have the patient open the eyes and immediately begin the reaching task. The starting position needs to be with the arm OUT OF VIEW AT THE MIDLINE. It is very important that the arms are not visible until they are reaching toward the target. Feeling around or trying to correct for reaching errors after the arms are visible needs to be avoided in order to get the full effect of the therapy.
- Using the right arm, instruct the patient to quickly reach and touch the target 100 times. The therapist should instruct the patient at random to the left or right targets. The clinician should watch for the expected reaching errors to the right of the target, with gradual correction over 10-20 trials.
- 100 targets should be achieved before moving on to the next step.
- If adaptation is not achieved even with 100 targets, continue until the patient appears adapted or becomes fatigued.
- Record number of targets and successful adaptation.
- The therapy should not be stopped short of 100 trials because the patient "appears adapted". Continued reaching maximizes the desired aftereffect

Step 3 Post Test and measurement of realignment:

- Have patient close eyes and not open until instructed to.
- Remove the prism glasses – keep eyes closed
- Have the patient open the eyes and immediately begin the reaching task again. The starting position needs to be with the arm OUT OF VIEW AT THE MIDLINE.
- The patient should miss left of the target. Note the magnitude of the initial miss in degrees.
- Note the number of trials to again reach the target accurately.

Other Considerations: The ideal therapeutic regimen is **2 sessions daily for 2 weeks**. Some patients will be unable to follow the protocol due to cognitive limitations or motor limitations. Patients who are unable to reach are unlikely to benefit from this therapy.

Protocol for rehabilitation of visuomotor coordination problems

Indications: Optic Ataxia, Cerebellar Ataxia, Oculomotor Apraxia, hemispatial neglect

Common Symptoms: Consistent Misreaching

Equipment Needed:
- Two or more targets

Frequency: Minimum of 3 times/week x 2 weeks

1. Repetitive t-VMT training (t-VMT= terminal[1] visuomotor task)
   a. 2 stickers on a table 1-2 in. right and left of midline, or with Dynavision or other similar visuo-motor board
      i. Occlude the patient's view of their hand and body in the starting position at the sternum
      ii. Have the patient reach quickly with the right hand to targets.
      iii. Bring the hand back and repeat for ~60 trials, randomly alternating between targets.
      iv. Repeat for the other hand if possible
      v. Repeat using common objects or incorporate into ADL training
2. Closed-loop visuomotor training (online corrections)
      i. Using a pipe cleaner shaped into a loop (adjusted diameter based on the ability of the patient).
      ii. Instruct the patient to make a pointer finger and keep their finger in the center of it as you move it.
      iii. Continue for 2 minutes
      iv. Repeat for the other hand if possible
3. Gaze-reach dissociation training
      i. Can be terminal reaching condition or open loop
      ii. Patient encouraged to reach for objects without foveating them

[1] Terminal Reaching (def). Hand is not visible until the end (terminus) of the reach.

3rd Oculomotor Neuro Re-education Protocol
(see manual for detailed instructions)

Frequency: A minimum of 3 sessions per week until goals are met or patient is discharged

Head-Rotations Single to Double

1. Red-green glasses may be used during the task to provide better feedback to the patient (but are not required)
2. Place a fixation target at distance (eg. small plus or "x")
3. Cue patient to notice double images
4. Alternate cover to break suppression if necessary (de-clutter or provide larger or brighter target- see manual for detailed anti-suppression techniques)
5. Slowly turn the head VERY SLOWLY to move the images closer together (find head position where it is single).
6. Slowly return the head to neutral, verbalizing when the target blurs or doubles (repeat for several minutes).
7. Repeat, going double to single
8. Repeat at far or near

***Try to incorporate this strategy during ADL's when the patient reports or is symptomatic of double vision.**

Tips: It usually takes 60-100 trials to achieve motor adaptation. If there is no perceived double vision, do the alternate-cover cover-uncover activity (see manual under anti-suppression activities). Also check that one eye is not closed or otherwise occluded.

Alternate Cover, Cover-Uncover:

1. Stabilize head.
2. Give a fixation target (eg. small plus or "x" at distance or near).
3. Cover one eye then the other. Watch for the eye to move to pick up fixation.
4. Alternate 15 times or more and then uncover both eyes (teach patient to use the cover).
5. Cue the patient to "look hard" or "focus" or "cross the eyes" to bring the images together. Provide sufficient time to try. Cue not to close an eye.
6. Repeat 3 times

Brock String or pencil push-ups

1) Fast Vergence (bead jumps): Repeated shift gaze
2) Slow Vergence (bug on string): Imagine bug walking down the string
3) Different positions of gaze: Primary gaze, down gaze, left gaze, right gaze

PROTOCOL FOR 4TH NERVE REHABILITATION

4th nerve oculomotor neuro re-education protocol (see manual for detailed instructions)

Frequency: A minimum of 3 sessions per week until goals are met or patient is discharged

1. Instruct patient to look at a small (~20/30) target such as a "+".
2. Very slowly tilt the head to the right shoulder and back to neutral, verbalizing when the target blurs, doubles, or crisscrosses.
3. Stop and slowly tip the head back towards the shoulder trying to bring the images together.
4. Once the images fuse, slowly tilt the head back towards neutral.
5. Repeat this for 5 minutes or until fatigue.
6. Repeat for double to single
7. Repeat for turn head side to side

***Try to incorporate this strategy during ADL's when the patient reports or is symptomatic of double vision.**

Tips: It usually takes 60-100 trials to achieve motor adaptation. If there is no perceived double vision, do the alternate-cover cover-uncover activity (see manual under anti-suppression activities). Also check that one eye is not closed or otherwise occluded.

6th Oculomotor Neuro Re-education Protocol
(see manual for detailed instructions)

Frequency: A minimum of 3 sessions per week

Head-Rotations Single to Double

1. Red-green glasses may be used during the task to provide better feedback to the patient (but are not required)
2. Place a fixation target at near (eg. small "+" or "x")
3. Cue patient to notice double images
4. Alternate cover to break suppression if necessary (de-clutter or provide larger or brighter target- see manual for detailed anti-suppression techniques)
5. Turn the head VERY SLOWLY to move the images closer together (find head position where it is single).
6. Slowly return the head to neutral, verbalizing when the target blurs or doubles (repeat for several minutes).
7. Repeat, going double to single
8. Repeat at far

***Try to incorporate this strategy during ADL's when the patient reports or is symptomatic of double vision.**

Alternate Cover, Cover-Uncover:

1. Stabilize head and give a fixation target (eg. small plus or "x" at near).
2. Cover one eye then the other. Watch for the eye to move to pick up fixation.
3. Alternate 15 times or more and then uncover both eyes
4. Cue the patient to "relax" or "look or think big and far" to bring the images together. Provide sufficient time to try. Cue not to close an eye.
5. Its ok if they can't fuse the images. It's important to practice trying.
6. Repeat 3 times

Thumb Saccades

1. Patch the non-paretic eye
2. Stabilize head
3. Hold thumbs shoulder width apart and look right to left 50 times or until fatigue

Brock String Divergence

1) Move the beads out of the way
2) Notice where the strings cross
3) Keep looking for at least 5 minutes continuously
4) Notice any changes in the position where the strings cross
5) Goal is to learn to activte divergence eye movements to get the area of crossing to shift further back (divergence).
6) Repeat in 10 degree left right and down gaze

PROTOCOL FOR BINOCULAR VISION REHABILITATION

A. Activities to promote fusion (seeing 1 image with both eyes open)

1. Alternate Cover, Cover-Uncover: Give a fixation target (eg. small plus or "x" at distance or near). Cover one eye then the other. Alternate several times and then uncover both eyes. Patient may see double when cover is taken away. Cue them to try to fuse the images by either focusing or relaxing. Provide sufficient time to try. Cue not to close an eye.
2. Pencil Push-up: Slowly bring a target towards the nose while keeping eyes aligned (look hard, focus!)
3. Prism Bar Vergences: Ramp up vergence demand at about 1 prism diopter per second or so, "keeping it single!"
4. Brock string
    a. jumps (fast vergence): Look at the red bead, look at the green bead, look at the yellow bead, repeat….
    b. Brock string bug on a string (slow vergence): "Slowly walk the "x" (crossed strings) towards the nose and away.
5. Prism Jump vergences (flippers or loose lens ok): Put base out on, fuse, take it off. Repeat….
6. Near-far saccades: Look to close target (it should be double for a second). Look hard, make it single. Now quickly to distance, same thing.

B. Transfer to ADL's

1. Using skills learned to promote fusion, attempt to transfer these skills during ADL training.
2. Safety Assessment: After 1-2 weeks with fusion exercises and prism (if needed), do trials comparing safety under monocular (patching) vs. binocular conditions (using head posturing and prism as necessary).

Protocol for Rehabilitation of Cortical Visual Impairment

Indications: Reduced visual acuity or problems with object and text identification attributable to cortical injury.

Examples of Conditions: Visual agnosias, Bilateral hemianopia, Simultagnosia

Equipment Needed:

1. Patient's glasses or loaner glasses	2. Black Marker Pen
3. White or off-white paper	4. Black construction paper
5. Goose neck lamp	6. Foam or block letters
7. Picture flash cards	8. Common household items
9. Report with minimum print size requirements	

Activities and modifications will depend on type and level of impairment. Listed here are the approved activities:
1. Letter identification with block letters
2. Trace and say letters and symbols
3. Word identification with block letters
4. Trace and say words
5. Identify objects (hold and touch)
6. Identify objects, flash cards. (error free learning)
7. Identify objects, flash cards (see and say)

Compensatory techniques: Train the patient to adapt their own environment to maximize performance

1. Contrast enhancement: Eg. black placemat, direct lighting with goose neck lamp, labels with large black on white or white on black print. Use symbols on the labels instead of words or letters, if this is easier.
2. Adaptive technology: Eg. reading scanners, CCTV, reader pen, ipad/pod/phone aps
3. Line and finger guides: Eg. fold or cut a window in construction paper to isolate objects of interest.
4. Tactile/auditory mods: tactile stickers (on phone, label 911 with tactile stickers), system for money identification (eg. 5's folded in half, right corner folded for 10's)
5. Anchoring and scanning to the worse side.

PROTOCOL FOR OCULOMOTOR REHABILITATION

Frequency: A minimum of 3 sessions per week until goals are met or patient is discharged

Indication: Vestibular-Oculo Reflex dysfunction, Saccadic Eye Movement Dysfunction, Pursuit Eye Movement Dysfunction

1. **Repetitive Voluntary Saccades**: Therapist or assistant stabilizes head patient's head. Patient hold thumbs up at width of ears (substitute other targets if hemiplegia). Right, left, right left: continue for ~60 trials, minimum of 3 times a week for 2 weeks.
2. **Gaze Stabilization:** (visual pursuit and VOR training)
   a. Head Rotations: Fixation "X" on patient's bulletin board (or other similar target). Patient slowly rotates head left-right-up-down-tilt left-tilt right. Begin seated, move to standing when possible. Continue for 5-10 minutes, a minimum of 3 times a week for 2 weeks. Perform 1 cycle during ADL training in attempt to transfer effect.
   b. Eye Rotations: Head stabilized, patient holds fixation stick (pen, popsicle stick, ect) and moves slowly (~3 deg/sec) left-right-up-down while trying to maintain fixation. Begin seated, move to standing when possible. Continue for 5-10 minutes, a minimum of 3 times a week for 2 weeks. Perform 1 cycle during ADL training in attempt to transfer effect.

Flowcharts for Postural Modifications

- Postural adaptations

3rd

- Right Eye Affected → Turn L → Distance Easier, Move Further
- Left Eye Affected → Turn R → Distance Easier, Move Further

*Cue to focus hard to eliminate diplopia

6th

- Right Eye Affected → Turn R → Move Closer
- Left Eye Affected → Turn L → Move Closer

*Cue to relax gaze to eliminate diplopia

4th

- Right Eye Affected → Bring reading material up &/or tilt chin in → Tilt head L → Turn head L
- Left Eye Affected → Bring reading material up &/or tilt chin in → Tilt head R → Turn head R

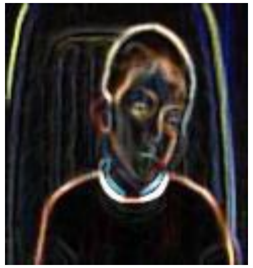

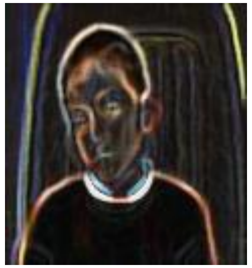

Supplement: Supplemental Appendix S1 [file mmc1.pdf]
