# Supplementary material for: Inpatient Virtual Vision Clinic Improves Access to Vision Rehabilitation Before and During the COVID-19 Pandemic
Source: Arch Rehabil Res Clin Transl. 2020 Dec 19;3(1):100100. doi: 10.1016/j.arrct.2020.100100 (PMC7749728; doi:10.1016/j.arrct.2020.100100)
Supplement: Supplemental Tables [file mmc2.docx]

Supplement Table S1

| *Unique contributions from each specialty in visual neuro-rehabilitation* | |
| --- | --- |
| Occupational Therapist (OT)  Visual Perceptual Deficits Specialist | Optometrist (OD)  Vision Rehabilitation Specialist |
| Observation of functional impairments | Provide ocular and visual diagnoses |
| Information from the initial visual/perceptual evaluation | Refractive Management |
| Impact of visual disorders on function | Prisms |
| Compensatory techniques and environmental adaptations for visual impairments | Restorative oculomotor therapies |
